# Prognostic value of combined LDL-C and remnant cholesterol stratification for 24-month major adverse cardiovascular events after primary PCI in patients with STEMI

**DOI:** 10.3389/fcvm.2026.1901661

**Published:** 2026-09-01

**Authors:** Qianying Xie, Chaoyi Wang, Wenjing Zhao, Lei Cai, Changjie Yu, Liting Pang

**Affiliations:** Department of Cardiology, Tiantai People’s Hospital of Zhejiang Province, Taizhou, China

**Keywords:** low-density lipoprotein cholesterol (LDL-C), major adverse cardiovascular events (MACE), percutaneous coronary intervention (PCI), remnant cholesterol (RC), ST-elevation myocardial infarction (STEMI)

## Abstract

**Objective:**

This single-center prospective observational cohort study aimed to investigate the prognostic value of combined low-density lipoprotein cholesterol (LDL-C) and remnant cholesterol (RC) stratification in patients with ST-elevation myocardial infarction (STEMI) undergoing primary percutaneous coronary intervention (PCI).

**Methods:**

A total of 812 STEMI patients were enrolled and stratified into four groups according to LDL-C and RC levels measured at 1-month post-PCI. The primary endpoint was 24-month major adverse cardiovascular events (MACE).

**Results:**

The overall MACE rate increased stepwise across the four subgroups (*P* < 0.001). Multivariate Cox regression showed that elevated RC was independently associated with MACE (HR = 1.559, 95% CI: 1.018−2.386, *P* = 0.041), whereas isolated high LDL-C was not (HR = 1.329, 95% CI: 0.992−1.781, *P* = 0.057). Compared with the low LDL-C + low RC group, the high LDL-C + high RC group carried the highest MACE risk (HR = 2.535, 95% CI: 1.591–4.041, *P* < 0.001), followed by the low LDL-C + high RC group (HR = 1.874, 95% CI: 1.153–3.045, *P* = 0.011).

**Conclusions:**

Combined LDL-C and RC stratification may assist risk evaluation in STEMI patients after primary PCI. Elevated RC, rather than isolated high LDL-C, is associated with adverse outcomes, which may help identify patients with residual lipid-related risk.

## Introduction

Acute ST-segment elevation myocardial infarction (STEMI) remains one of the most life-threatening acute coronary syndromes, characterized by rapid onset, extensive myocardial necrosis, and high short-term mortality. Primary percutaneous coronary intervention (PCI) has become the gold-standard reperfusion strategy for STEMI, effectively restoring epicardial blood flow, salvaging ischemic myocardium, and reducing in-hospital mortality (1, 2). Despite guideline-directed medical therapy including dual antiplatelet therapy, high-intensity statin-based lipid-lowering treatment, *β*-blockers, and renin–angiotensin–aldosterone system inhibitors, a substantial proportion of patients still experience major adverse cardiovascular events (MACE) within 1 to 3 years after successful PCI, such as recurrent myocardial infarction, ischemic stroke, heart failure re-hospitalization, and cardiovascular death (3–5). Although intensive LDL-C lowering therapy effectively reduces cardiovascular events, recurrent ischemic risk persists in many treated patients, suggesting additional lipid-mediated atherogenic burden independent of LDL-C may contribute to residual cardiovascular risk.

Remnant cholesterol (RC), defined as the cholesterol content of triglyceride-rich lipoprotein remnants including very-low-density lipoprotein, intermediate-density lipoprotein, and chylomicron remnants, has emerged as a critical and independent atherogenic lipid marker beyond LDL-C (5, 6). Unlike LDL-C, RC may be unrestrictedly engulfed by macrophages through scavenger receptors. Prior mechanistic studies suggest that this process could facilitate foam cell generation and the development of vulnerable atherosclerotic plaques. Mounting epidemiological and clinical evidence has confirmed that elevated RC is strongly associated with an increased risk of incident cardiovascular events, even in patients with well-controlled LDL-C levels (7–9). Recently, the concept of combined LDL-C and RC stratification has been proposed to improve risk stratification; this integrated classification stratifies individuals into four subgroups according to whether LDL-C and RC are simultaneously normal or elevated, providing a more comprehensive reflection of overall atherogenic burden than either marker alone (6, 10, 11).

Although the predictive value of combined LDL-C and RC stratification has been validated in elderly hypertensive patients, stable coronary artery disease, and dysglycemic populations, data specifically focusing on post-PCI STEMI patients—an extremely high-risk cohort with intense atherothrombotic stress—remain scarce. Furthermore, most available real-world evidence originates from large urban tertiary medical centers. Complementary validation data from county-level community hospitals are limited, which restricts the generalizability of this lipid stratification tool to broader grassroots clinical populations (12–16). Therefore, we designed this prospective cohort study to investigate the association between combined LDL-C and RC stratification and postprocedural MACE in STEMI patients undergoing PCI. We hypothesized that an inconsistent or elevated LDL-C/RC profile is independently predictive of adverse cardiovascular outcomes and that this combined classification can enhance risk stratification beyond conventional lipid parameters, supporting personalized lipid-lowering strategies in real-world clinical care.

Compared with prior RC-related studies in STEMI or ACS populations, our research has three core innovations. First, unlike most existing evidence originating from large urban tertiary hospitals, this study delivers real-world data from a county-level grassroots hospital to extend the generalizability of lipid stratification tools. Second, we measured lipid profiles one month after PCI to eliminate confounding acute-phase lipid fluctuations, which enhances the stability of risk grouping compared with studies using admission blood lipids. Third, we applied combined LDL-C and RC four-category stratification, and further verified that elevated RC, instead of isolated high LDL-C, dominates residual cardiovascular risk in post-PCI STEMI patients (5, 10).

## Methods

### Study design

This was a single-center, prospective, observational cohort study conducted at the Department of Cardiology, Tiantai People's Hospital, Taizhou, Zhejiang, China. The study protocol was approved by the Ethics Committee of Tiantai People's Hospital and was performed in accordance with the Declaration of Helsinki. All participants provided written informed consent before enrollment.

### Study population

All sequential patients admitted with STEMI who received emergency primary PCI at Tiantai People's Hospital from July 2022 to June 2024 were initially screened consecutively. We applied prespecified inclusion and exclusion criteria to this unselected consecutive admission cohort, and only individuals satisfying all eligibility standards were finally included in the analysis. No selective exclusion of eligible patients was performed during enrollment.

### Inclusion criteria

1. Diagnosis of STEMI consistent with the Fourth Universal Definition of Myocardial Infarction.
2. Primary PCI performed within 12 h of symptom onset, with post-procedural Thrombolysis in Myocardial Infarction (TIMI) flow grade 3.
3. Age ≥ 18 years, regardless of gender.
4. Complete baseline lipid profiles and ability to comply with scheduled follow-up.

### Exclusion criteria

1. Previous history of myocardial infarction, PCI, or coronary artery bypass grafting (CABG).
2. Acute stroke, transient ischemic attack, or severe peripheral vascular disease within 3 months before admission.
3. Severe hepatic or renal dysfunction, malignant tumor, coagulation disorders, or active bleeding.
4. Thyroid dysfunction, familial hypercholesterolemia, or other diseases affecting lipid metabolism.
5. Unavailable lipid data or calculated remnant cholesterol (RC) ≤ 0 mmol/L.
6. Expected survival < 2 year or high risk of loss to follow-up.

### Classification of combined LDL-C and RC stratification

To avoid the acute-phase interference of STEMI on lipid levels, LDL-C and RC were measured at 1 month after PCI using fasting venous blood samples (17). Complete admission lipid data and serial follow-up lipid tests were unavailable, so we could not compare lipid levels at admission versus 1-month post-PCI or assess the prognostic effect of longitudinal lipid changes.

Serum total cholesterol (TC), high-density lipoprotein cholesterol (HDL-C), triglycerides (TG) were detected via standard enzymatic colorimetric assays on an automatic biochemical analyzer. LDL-C was measured using the direct homogeneous assay method. Notably, remnant cholesterol (RC) was not directly measured by the instrument; it was mathematically calculated with the formula:

RC = Total cholesterol (TC)−LDL-C−high-density lipoprotein cholesterol (HDL-C) (18).

According to the 2019 ESC/EAS Dyslipidemia Guidelines and validated cutoff values for high-risk populations, patients were stratified into four groups (19, 20):

1. Low LDL-C + Low RC: LDL-C < 1.8 mmol/L and RC < 0.62 mmol/L
2. Low LDL-C + High RC: LDL-C < 1.8 mmol/L and RC ≥ 0.62 mmol/L
3. High LDL-C + Low RC: LDL-C ≥ 1.8 mmol/L and RC < 0.62 mmol/L
4. High LDL-C + High RC: LDL-C ≥ 1.8 mmol/L and RC ≥ 0.62 mmol/L

This LDL-C cutoff of 1.8 mmol/L (70 mg/dL) was derived from the 2019 ESC/EAS dyslipidaemia guidelines, the standard adopted during patient enrollment.

The RC cutoff of 0.62 mmol/L originates from large European ASCVD cohorts and is validated for risk stratification in post-PCI STEMI patients. No official guideline-defined therapeutic target exists for RC, yet multiple real-world studies prove RC ≥0.62 mmol/L predicts higher long-term cardiovascular risk in STEMI patients despite optimal LDL-C control. We adopted this threshold to screen patients with residual lipid risk (5, 10).

Fasting blood lipids were collected 1 month after PCI for risk stratification. Patients who experienced MACE or all-cause death prior to this lipid assessment were excluded, since valid 1-month lipid values could not be obtained to complete grouping.

## Data collection

### Baseline characteristics

Age, gender, body mass index (BMI), smoking status, hypertension, diabetes mellitus, prior stroke, atrial fibrillation, and baseline medications were recorded.

### Laboratory parameters

Glycated hemoglobin (HbA1c), serum creatinine, B-type natriuretic peptide (BNP), were measured using standard automated biochemical assays in the clinical laboratory of Tiantai People's Hospital. Lipid parameters used for risk stratification were obtained at 1 month after PCI to avoid acute-phase interference.

### Procedural data

Infarct-related artery, number of diseased vessels（≥50% stenosis）, stent number and dimensions were documented.

### Follow-up and study endpoints

Patients were followed up every 3 months for up to 24 months after discharge via outpatient review, telephone contact, or electronic medical record query at Tiantai People’s Hospital.

The endpoint was major adverse cardiovascular events (MACE), a composite of cardiovascular death, nonfatal recurrent myocardial infarction, ischemic stroke, and heart failure–related re-hospitalization. All endpoints were adjudicated blindly by two independent cardiologists; any disagreements were resolved by a senior cardiologist.

A total of 7 patients were lost to follow-up within the 24-month observation period, yielding an overall follow-up completion rate of 99.1% (805/812). All participants who failed to complete full follow-up were treated as censored observations in Kaplan–Meier and Cox regression analyses.

### Sample size estimation

Sample size estimation was performed before study initiation based on the primary endpoint of 24-month major adverse cardiovascular events (MACE). According to previous studies and real-world data, the 24-month MACE rate was assumed to be 13% in the low LDL-C + low RC group and 32% in the high LDL-C + high RC group. With a two-sided type I error of 0.05 and a statistical power of 80%, a minimum of 160 subjects per group was required. Considering a 10% expected loss to follow-up, the total target sample size was determined to be approximately 800 patients to ensure sufficient statistical power for between-group comparisons.

The assumed 24-month MACE rates for sample size calculation were derived from published real-world STEMI cohorts focusing on remnant cholesterol-related cardiovascular risk (5, 21).

### Statistical analysis

Statistical analyses were performed using SPSS 27.0. A two-sided *P* < 0.05 was considered statistically significant. Continuous variables were presented as mean ± standard deviation (SD) or median (interquartile range, IQR) and compared using Student's test, ANOVA, or nonparametric tests. Categorical variables were presented as counts (percentages) and compared using the chi-square test. Kaplan–Meier curves were constructed for cumulative MACE incidence, and differences were tested using the log-rank test. Univariate and multivariate Cox proportional hazards regression were used to identify independent predictors of MACE.

The proportional hazards (PH) assumption for Cox regression was verified before multivariate analysis by testing time-dependent covariates and log-minus-log survival plots. All variables satisfied the PH assumption, so the Cox proportional hazards regression model was applicable in this study.

We assessed lipid marker collinearity by VIF. Owing to the mathematical formula of RC, TC and HDL-C were removed from the multivariate Cox model to mitigate severe collinearity. Only LDL-C and RC remained, with VIF < 10 for both variables.

## 4 Results

### 4.1 Baseline characteristics

Between July 2022 and June 2024, a total of 870 consecutive STEMI patients who underwent primary PCI at Tiantai People’s Hospital were initially screened.

Among them, 58 patients were excluded for the following reasons: prior myocardial infarction/PCI/CABG (*n* = 16); severe liver/renal dysfunction, malignant tumor or coagulation disorders (*n* = 12); thyroid disease, familial hypercholesterolemia and other lipid metabolism disorders (*n* = 11); death or MACE occurring before the 1-month lipid assessment (*n* = 6); missing lipid data or incalculable RC (*n* = 9); expected survival less than 2 years and high loss-to-follow-up risk (*n* = 4). Ultimately, 812 patients were enrolled for final analysis.

The mean age was 63.8 ± 9.6 years, and 73.0% (593/812) were male.

According to lipid levels measured at 1 month after PCI, patients were stratified into four groups:

- Low LDL-C + Low RC (*n* = 226, 27.8%)
- Low LDL-C + High RC (*n* = 179, 22.0%)
- High LDL-C + Low RC (*n* = 238, 29.3%)
- High LDL-C + High RC (*n* = 169, 20.8%)

Table 1 presents the baseline clinical, laboratory, and medication data of the 812 included patients, stratified by combined LDL-C and RC levels. Significant intergroup differences were observed in age, diabetes mellitus, HbA1c, Scr, TC, LDL-C, HDL-C, TG, and RC (all *P* < 0.05). In contrast, BMI, smoking status, atrial fibrillation, BNP, LVEF, LVESD, and LVEDD were comparable among the four subgroups (all *P* > 0.05). Regarding medications, statin use was uniformly high and well balanced. The utilization of fenofibrate, ezetimibe, *β*-blockers, and RAAS inhibitors also showed no significant between-group differences (all *P* > 0.05).

**Table 1:** Baseline characteristics.

Variable	Low LDL C + Low RC (*n* = 226)	Low LDL C + High RC (*n* = 179)	High LDL C + Low RC (*n* = 238)	High LDL C + High RC (*n* = 169)	*P* value
Demographic characteristics					
Age, years	61.2 ± 8.7	64.5 ± 9.2	63.1 ± 9.5	66.8 ± 10.1	<0.001
Male, *n* (%)	168 (74.3)	127 (70.9)	176 (73.9)	122 (72.2)	0.861
BMI, kg/m^2^	24.5 ± 3.0	24.1 ± 3.2	24.3 ± 3.1	24.0 ± 3.3	0.396
smoker, *n* (%)	97 (42.9)	71 (39.7)	99 (41.6)	61 (36.1)	0.556
Comorbidities					
Hypertension, *n* (%)	121 (53.5)	102 (56.4)	142 (59.7)	87 (51.5)	0.363
Diabetes mellitus, *n* (%)	56 (24.8)	58 (32.4)	67 (28.2)	64 (37.9)	0.033
Atrial fibrillation, *n* (%)	13 (5.8)	14 (7.8)	16 (6.7)	13 (7.7)	0.831
Laboratory parameters					
HbA1c, %	5.7 ± 0.7	5.9 ± 0.9	5.8 ± 0.8	6.2 ± 0.9	<0.001
Scr, μmol/L	88.4 ± 18.7	90.4 ± 21.8	87.6 ± 21.0	95.2 ± 22.6	0.002
BNP, pg/mL	152.9 ± 49.1	148.9 ± 53.1	150.0 ± 57.8	147.3 ± 54.3	0.761
TC, mmol/L	3.52 ± 0.77	3.99 ± 0.89	4.57 ± 1.01	4.82 ± 1.09	<0.001
LDL-C, mmol/L	1.25 ± 0.26	1.38 ± 0.25	2.28 ± 0.31	2.38 ± 0.33	<0.001
HDL-C, mmol/L	1.19 ± 0.29	1.00 ± 0.19	1.10 ± 0.29	0.99 ± 0.20	<0.001
TG, mmol/L	1.51 ± 0.69	1.38 ± 0.70	1.57 ± 0.62	1.62 ± 0.67	0.006
RC, mmol/L	0.35 ± 0.16	0.97 ± 0.21	0.42 ± 0.13	1.06 ± 0.30	<0.001
LVEF, %	53.3 ± 8.2	51.6 ± 8.3	52.4 ± 8.3	51.3 ± 9.4	0.090
LVESD (mm)	35.9 ± 5.0	36.7 ± 8.4	35.2 ± 5.9	36.2 ± 6.0	0.092
LVEDD (mm)	47.2 ± 4.4	48.0 ± 4.9	47.4 ± 3.8	47.2 ± 5.4	0.206
medications, *n* (%)					
Statin	219 (96.9)	173 (96.6)	231 (97.1)	163 (96.4)	0.987
Fenofibrate	40 (17.7)	41 (22.9)	45 (18.9)	39 (23.1)	0.426
Ezetimibe	26 (11.5)	24 (13.4)	31 (13.0)	28 (16.6)	0.535
*β*-blocker	115 (50.9)	108 (60.3)	127 (53.4)	90 (53.3)	0.279
RAAS inhibitor	97 (42.9)	82 (45.8)	96 (40.3)	64 (37.9)	0.461

Of note, these between-group disparities in key risk factors were predominantly seen in subgroups with elevated RC, which had older age, higher prevalence of diabetes mellitus, higher HbA1c and Scr, and a greater proportion of three-vessel coronary artery disease.

### 4.2 Angiographic and procedural characteristics

As summarized in Table 2, the distribution of infarct-related artery was similar across the four lipid subgroups (*P* = 0.497). The number of diseased vessels differed significantly (*P* = 0.031), with a higher proportion of three-vessel disease observed in the high-lipid subgroups. Symptom-to-balloon time, no-reflow/slow-reflow phenomenon, number of implanted stents, mean stent diameter, and mean stent length were comparable among groups (all *P* > 0.05).

**Table 2:** Angiographic and procedural features.

Variable	Low LDL C + Low RC (*n* = 226)	Low LDL C + High RC (*n* = 179)	High LDL C + Low RC (*n* = 238)	High LDL C + High RC (*n* = 169)	*P* value
Infarct related artery, *n* (%)					0.497
Left anterior descending artery	128 (56.6)	82 (45.8)	124 (52.1)	87 (51.5)	
Right coronary artery	62 (27.4)	59 (33.0)	70 (29.4)	78 (32.8)	
Left circumflex artery	36 (15.9)	38 (21.2)	44 (18.5)	35 (20.7)	
Number of diseased vessels, *n* (%)					0.031
Single-vessel disease	128 (56.6)	93 (52.0)	107 (45.0)	69 (40.8)	
Two-vessel disease	62 (27.4)	55 (28.5)	76 (31.9)	56 (33.1)	
Three-vessel disease	36 (15.9)	31 (17.3)	55 (23.1)	44 (26.0)	
Symptom to balloon time, min	252.2 ± 75.4	257.3 ± 79.1	248.2 ± 78.8	265.8 ± 83.8	0.150
No reflow/slow reflow, *n* (%)	12 (5.3)	13 (7.3)	15 (6.3)	14 (8.3)	0.674
Number of implanted stents	1.2 ± 0.4	1.3 ± 0.5	1.2 ± 0.4	1.4 ± 0.5	0.186
Mean stent diameter, mm	3.00 ± 0.33	2.99 ± 0.29	3.03 ± 0.31	2.98 ± 0.31	0.625
Mean stent length, mm	23.5 ± 5.4	23.9 ± 5.8	23.2 ± 5.1	24.4 ± 5.6	0.156

### 4.3 Incidence of 24-month MACE

Kaplan–Meier analysis revealed significant intergroup differences in the cumulative incidence of overall MACE (log-rank *P* < 0.001). Nevertheless, no statistically significant intergroup differences were observed for individual endpoint components, including cardiovascular death, recurrent myocardial infarction, ischemic stroke, and heart failure–related rehospitalization (Table 3).

**Table 3:** Incidence of MACE at 24-month follow-up.

Event	Low LDL-C + Low RC (*n* = 226)	Low LDL-C + High RC (*n* = 179)	High LDL-C + Low RC (*n* = 238)	High LDL-C + High RC (*n* = 169)	*P* value
MACE(Total)	28 (12.4%)	39 (21.8%)	41 (17.2%)	48 (28.4%)	<0.001
MACE (components)					
Cardiovascular death	6 (2.7%)	9 (5.0%)	8 (3.4%)	11 (6.5%)	0.230
recurrent myocardial infarction	7 (3.1%)	10 (5.6%)	11 (4.6%)	13 (7.7%)	0.216
Ischemic stroke	3 (1.3%)	4 (2.2%)	4 (1.7%)	5 (3.0%)	0.681
Heart failure–related rehospitalization	12 (5.3%)	16 (8.9%)	18 (7.6%)	19 (11.2%)	0.179

### 4.4 Kaplan–Meier survival analysis

**Figure 1:**
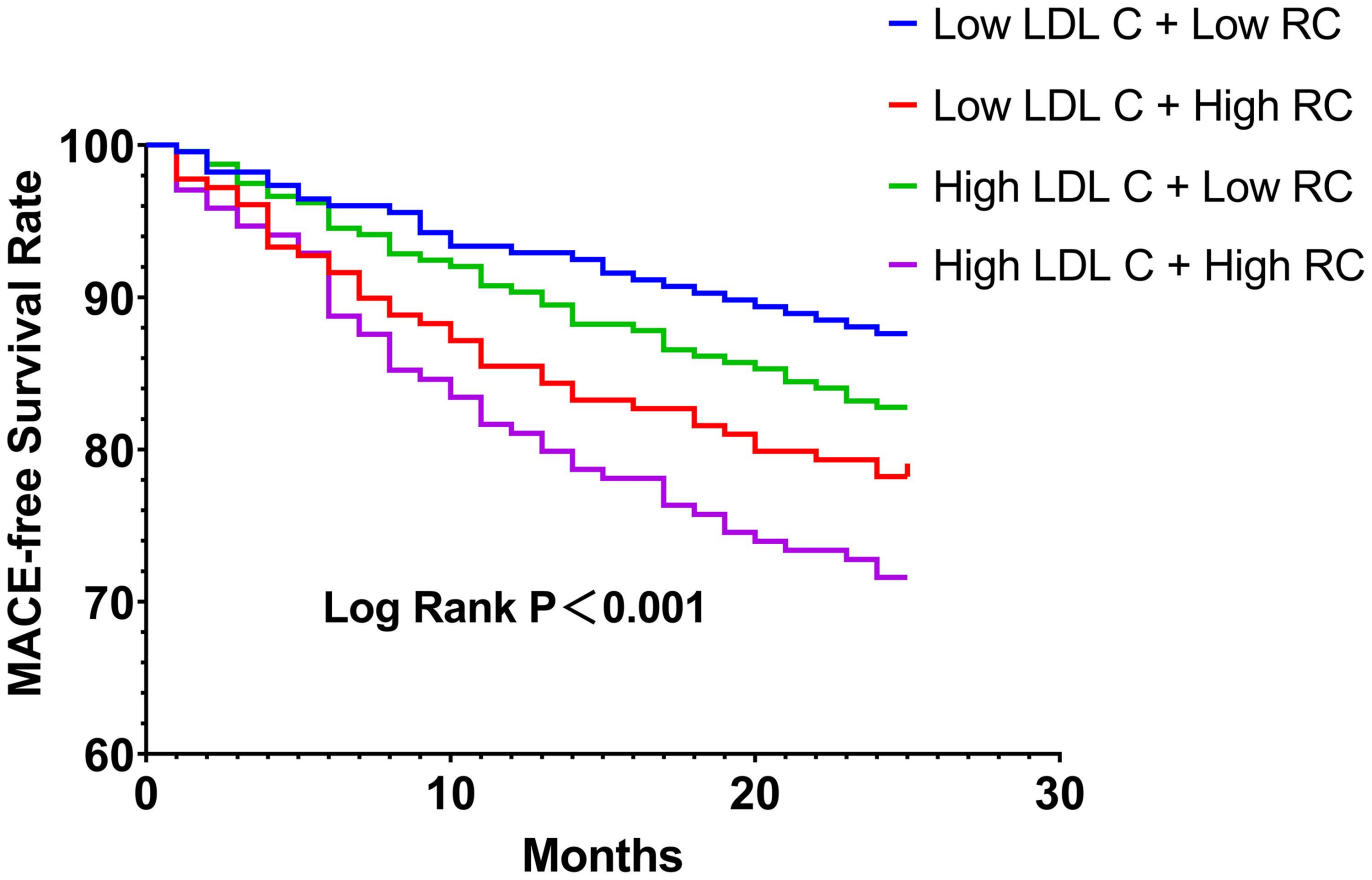
MACE-free survival according to lipid stratification.

Kaplan–Meier analysis was performed to compare 24-month MACE-free survival across the four lipid-stratified subgroups (Figure 1). Patients in the Low LDL-C + Low RC group showed the highest rate of MACE-free survival throughout the follow-up period, while those in the High LDL-C + High RC group experienced the steepest decline in event-free survival. The Low LDL-C + High RC and High LDL-C + Low RC groups showed intermediate survival trajectories, with the former exhibiting a slightly lower survival rate than the latter. The differences in MACE-free survival among the four groups were statistically significant (log-rank *P* < 0.001).

### 4.5 Cox regression analysis

Univariate and multivariate Cox regression analyses were performed to identify independent predictors of 24-month MACE (Table 4). In univariate analysis, LDL-C, HDL-C, and RC were significantly associated with MACE. After multivariate adjustment, RC remained an independent predictor (HR = 1.559, 95% CI: 1.018−2.386, *P* = 0.041), whereas LDL-C showed only a borderline association (HR = 1.329, 95% CI: 0.992−1.781, *P* = 0.057).

**Table 4:** Univariate and multivariate Cox regression for 24-month MACE.

Variables	Univariate analysis	*P* value	Multivariate analysis	*P* value
	HR (95% CI)		HR (95% CI)	
Age	1.008 (0.992–1.025)	0.330	1.003 (0.987–1.020)	0.688
Diabetes mellitus	1.067 (0.754–1.508)	0.715	1.129 (0.796–1.600)	0.497
HbA1c, %	1.220 (1.010–1.474)	0.039	1.154 (0.953–1.396)	0.143
Scr	1.000 (0.992–1.007)	0.935	0.998 (0.991–1.006)	0.670
TC	1.033 (0.894–1.193)	0.659		
LDL-C, mmol/L	1.363 (1.044–1.781)	0.023	1.329 (0.992–1.781)	0.057
HDL-C,mmol/L	0.510 (0.277–0.941)	0.031		
TG, mmol/L	1.045 (0.827–1.319)	0.714	1.021 (0.805–1.294)	0.866
RC, mmol/L	1.788 (1.201–2.662)	0.004	1.559 (1.018–2.386)	0.041
Three vessel disease	1.084 (0.634–1.468)	0.817	—	—

In the fully-adjusted Cox regression model with continuous lipid variables, the cross-product term of LDL-C and RC showed no significant multiplicative interaction (*P* = 0.936, HR = 0.971, 95% CI: 0.476–1.981). For additive interaction assessment, we dichotomized LDL-C and RC using predefined cutoffs (LDL-C ≥ 1.8 mmol/L, RC ≥ 0.62 mmol/L). The fully-adjusted hazard ratios were 1.409 for isolated elevated LDL-C, 1.679 for isolated elevated RC, and 2.216 for concurrent elevation of both lipids. Additive interaction indices were calculated as follows: RERI = 0.127 (95% CI: −1.079 to 1.145), AP = 0.057, and synergy index = 1.117. Since the 95% CI of RERI crossed zero, no statistically significant additive synergistic interaction between LDL-C and RC was identified in this STEMI cohort.

### 4.6 Prognostic impact of lipid stratification

**Figure 2:**
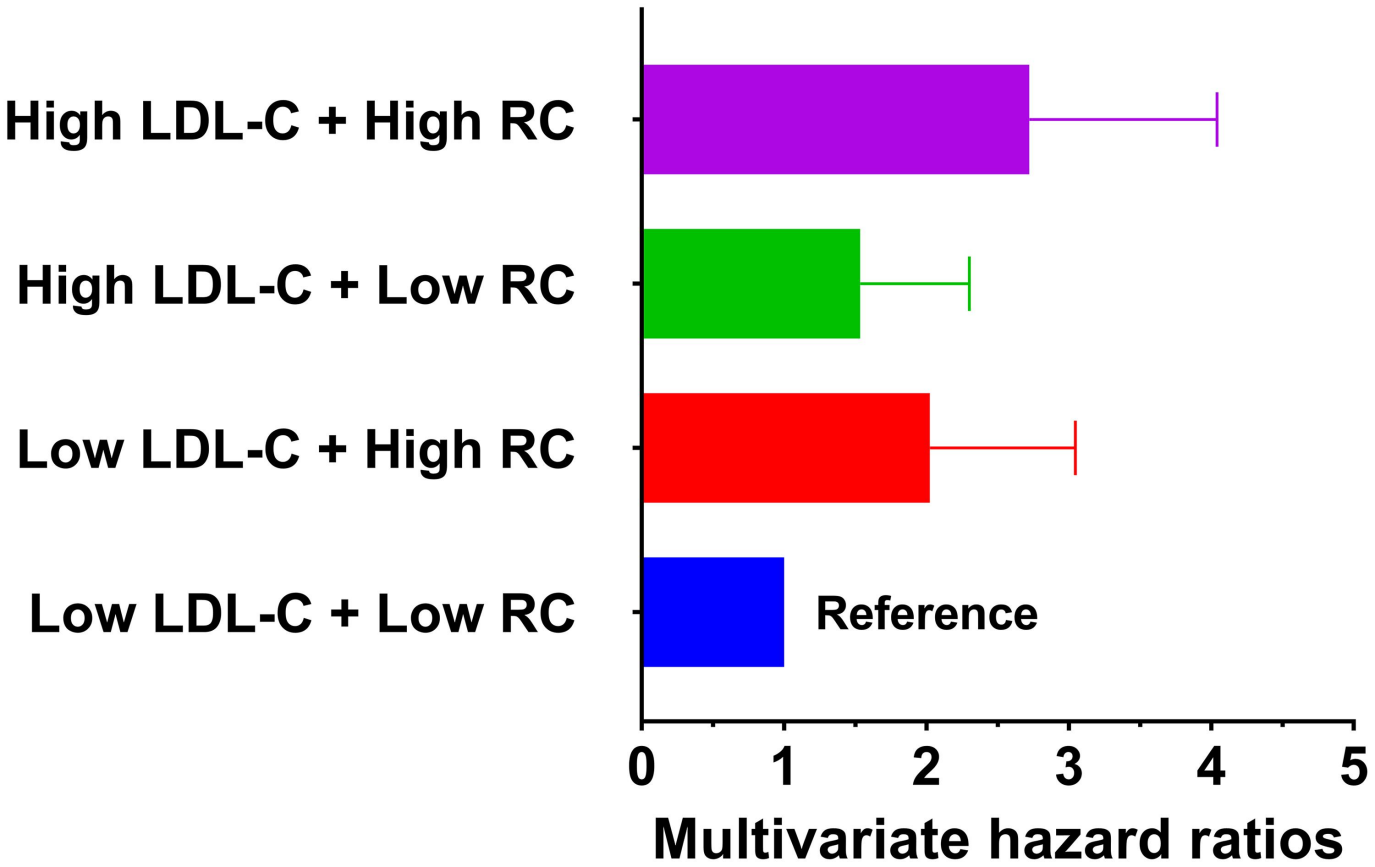
Adjusted hazard ratios of lipid subgroups.

Multivariate Cox regression analysis was further conducted using the Low LDL-C + Low RC group as the reference (Figure 2). Compared with the reference group, the Low LDL-C + High RC group exhibited a significantly higher risk of MACE (HR = 1.874, 95% CI: 1.153–3.045, *P* = 0.011). The High LDL-C + Low RC group showed an increased but non significant trend (HR = 1.423, 95% CI: 0.880–2.300, *P* = 0.150). The highest MACE risk was observed in the High LDL-C + High RC group (HR = 2.535, 95% CI: 1.591–4.041, *P* < 0.001).

### 4.7 ROC analysis of baseline RC and LDL-C for 24-month MACE

**Figure 3:**
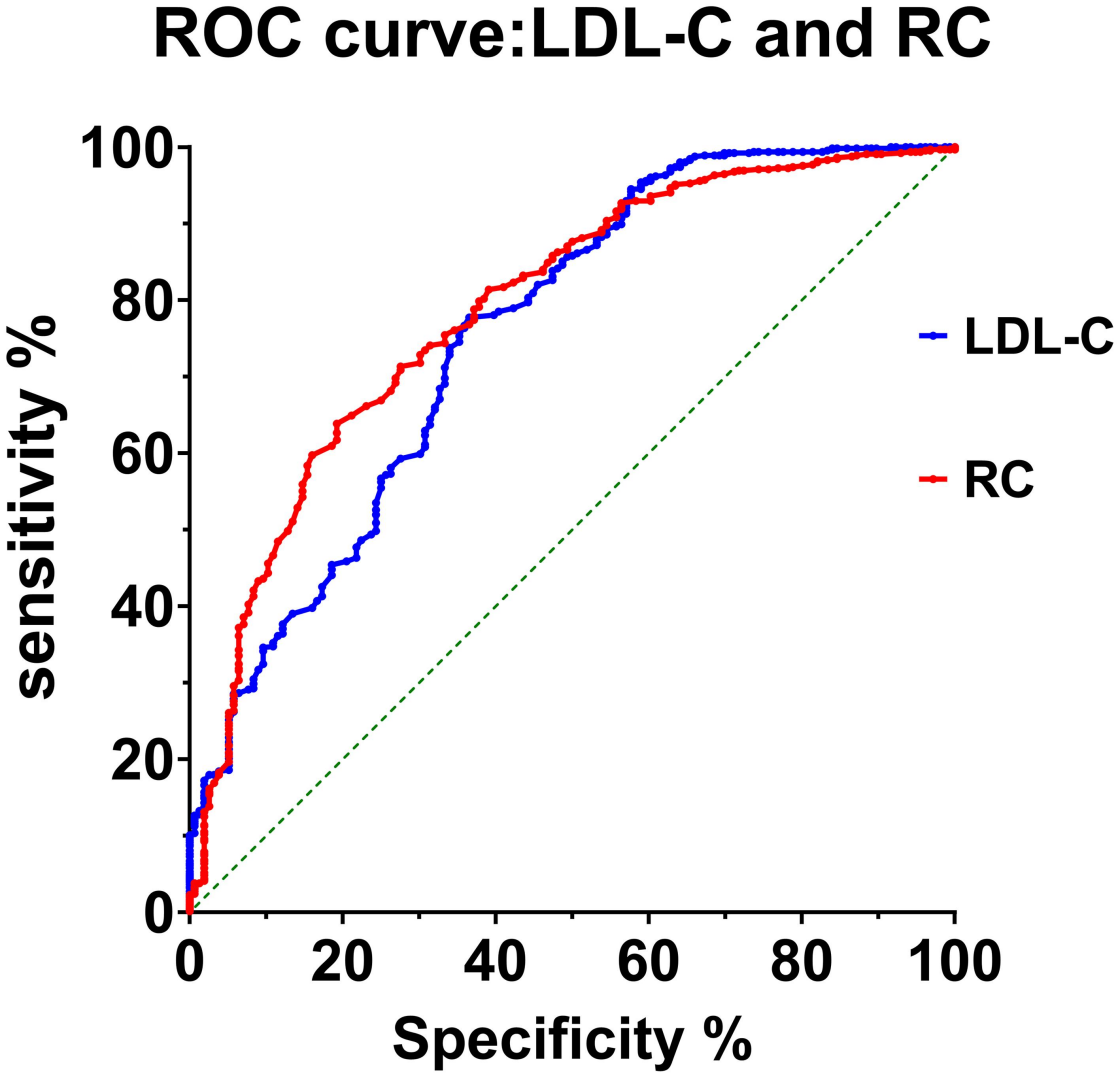
ROC curves for baseline LDL-C and remnant cholesterol (RC) to predict 24-month MACE.

Receiver operating characteristic curve analysis (Figure 3) was performed for prediction of 24-month MACE. The AUC for baseline RC was 0.789 (95% CI 0.748–0.829, *P* < 0.001), and the AUC for baseline LDL-C was 0.757 (95% CI 0.712–0.802, *P* < 0.001).

## 5 Discussion

This single-center prospective observational cohort study enrolled STEMI patients receiving primary PCI. As an observational analysis, it can only identify associations rather than confirm causal links between lipid profiles and cardiovascular events. Two key findings were obtained. First, combined LDL-C and RC stratification showed a stepwise gradient of 24-month MACE risk; patients with concurrent elevated LDL-C and RC had the highest event risk. After multivariate adjustment, elevated RC independently correlated with higher MACE risk even in patients with target LDL-C, while isolated high LDL-C had no significant correlation with adverse outcomes. Second, MACE risk rose progressively across the four lipid subgroups, indicating incremental prognostic value of this combined stratification. These results support the clinical value of integrated LDL-C and RC management to improve post-PCI prognosis in STEMI patients.

In recent years, LDL-C has been recognized as the primary therapeutic target for cardiovascular risk reduction. However, the concept of “residual cardiovascular risk” despite LDL-C lowering has gained increasing attention, and remnant cholesterol (RC) has emerged as a key contributor to this risk. RC refers to the cholesterol content contained in triglyceride-rich atherogenic remnant lipoproteins, including very low-density lipoprotein (VLDL) and intermediate-density lipoprotein (IDL) (19, 20). Mechanistically, these remnant particles are thought to induce endothelial dysfunction and vascular inflammation, thereby promoting atherosclerotic progression and plaque instability. In the setting of STEMI, where systemic inflammation is heightened and atherosclerotic burden is severe, elevated RC may further augment cardiovascular risk and predispose patients to recurrent adverse events (22, 23).

While both LDL-C and RC are associated with cardiovascular risk, their combined use may provide a more refined risk stratification strategy than either alone. In our study, the four lipid subgroups exhibited a clear stepwise increase in MACE-free survival risk: patients with both low LDL-C and low RC had the most favorable outcomes, followed by those with isolated high LDL-C, isolated high RC, and finally those with both elevated LDL-C and RC. Notably, even patients with LDL-C within the recommended target range but elevated RC had a significantly higher risk of adverse events. This combined stratification may have practical implications in clinical practice. Prior randomized controlled trials have reported that fibrates and omega-3 fatty acids may reduce triglyceride and remnant cholesterol levels (24). Intensive combined lipid-lowering regimens also show potential to mitigate lipid burden in hyperlipidemic populations. However, the present observational study only reveals associations between elevated RC and cardiovascular events and lacks interventional data to confirm whether RC-targeted treatments improve long-term prognosis. Clinical application of these lipid-lowering strategies requires validation from dedicated randomized trials. Furthermore, the identification of the highest-risk subgroup (high LDL-C + high RC) allows for targeted interventions and closer clinical follow-up, which may help reduce the burden of recurrent events in this vulnerable population (22, 24–26).

Our findings are consistent with and extend previous reports on the prognostic significance of RC in patients with coronary artery disease. Several observational studies and meta-analyses have demonstrated that elevated RC is independently associated with increased risk of cardiovascular events, even in patients treated with statins and with well-controlled LDL-C (6, 25). The present study adds to this body of evidence by focusing specifically on patients with STEMI undergoing primary PCI, a population at particularly high risk of recurrent events (22, 23).

Notably, in contrast to some previous studies, we found that isolated high LDL-C was not independently associated with MACE after adjustment for RC and other confounders (22, 27). This discrepancy may be partly explained by differences in study populations, the timing of lipid measurement, and the use of different adjustment variables. In our study, lipid levels were measured at one month after the index event, a time point chosen to avoid the transient lipid-lowering effect of the acute phase response, which may have provided a more accurate reflection of the patient's baseline lipid profile. Additionally, the inclusion of multiple clinical and procedural covariates in the multivariate model may have attenuated the association between LDL-C alone and outcomes, highlighting the stronger independent prognostic value of RC in this cohort.

The present study has several strengths. First, lipid levels were measured at one month post-PCI, thereby avoiding the confounding effects of acute-phase changes on lipid parameters. Second, the use of combined LDL-C and RC stratification allowed for a more comprehensive assessment of lipid-related risk, and the multivariate Cox regression model was adjusted for relevant clinical and procedural variables to minimize confounding. Finally, the 24-month follow-up period and the use of standardized definitions for MACE ensure the reliability of the outcome data.

This study has several limitations that warrant discussion. First, as a single-center observational study, our findings have limited generalizability, and causal inferences cannot be drawn. Second, although we adjusted for multiple confounders, residual confounding from unmeasured factors (e.g., medication adherence, genetic predisposition) could not be fully excluded. Third, we did not measure other lipid parameters such as lipoprotein(a) or apolipoprotein B, which may also contribute to residual cardiovascular risk. Fourth, the relatively small sample size in some subgroups may have limited statistical power to detect significant associations, particularly in the isolated high LDL-C group. Fifth, all lipid measurements were obtained at 1-month post-PCI. Patients suffering MACE or death within the first month were excluded due to unavailable lipid data. Since the 1-month lipid profiles were used to predict events starting from the index PCI, potential lead-time bias could exist. Sixth, we only collected basic types of lipid-lowering drugs, lacking records of statin intensity, medication adjustments, long-term adherence and PCSK9 inhibitor use, precluding relevant subgroup analyses and introducing potential confounding. Besides, lipids were only tested once at 1-month post-PCI without serial sampling at admission or follow-up visits; we could not assess cumulative RC burden or longitudinal lipid trajectories, which limits the novelty of this study. Seven, patient stratification relied on the 1.8 mmol/L LDL-C cutoff from the 2019 ESC/EAS guidelines. Newer guidelines propose a stricter LDL-C target for very high-risk STEMI patients. Since this updated standard was published after our patient enrollment, we were unable to adjust grouping criteria in advance. Adoption of the lower threshold may change subgroup composition and event risk associations, which limit direct extrapolation of our stratification strategy to current clinical practice.

Eighth, we did not record data on complete versus incomplete revascularization, or immediate versus staged PCI for patients with multivessel coronary disease. Staged revascularization may affect long-term MACE risk, and the lack of these detailed procedural parameters constitutes an unmeasured confounding factor in our analysis.

Ninth, we did not systematically collect detailed antiplatelet subtypes or PCSK9 inhibitor usage at baseline, which precluded subgroup analyses stratified by these antithrombotic and novel lipid-lowering therapies. Unmeasured differences in antiplatelet regimens and PCSK9 treatment may introduce residual confounding to our risk association results.

Future research should address several important aspects. Multicenter prospective studies with larger sample sizes are warranted to validate the prognostic value of combined LDL-C and RC stratification. In addition, clinical trials investigating the efficacy of RC-lowering strategies on cardiovascular outcomes, especially in STEMI patients with controlled LDL-C, are of great clinical importance.

## Conclusions

In conclusion, this prospective observational cohort study may provide preliminary associative evidence that combined LDL-C and RC stratification correlates with 24-month MACE risk in STEMI patients after primary PCI. After multivariable adjustment, elevated RC, rather than isolated high LDL-C, was associated with increased 24-month MACE risk. These findings may imply that RC could serve as a potential marker reflecting residual cardiovascular risk. Further large-scale multicenter studies and interventional trials are needed to validate the clinical utility of RC and explore whether RC-targeted lipid management benefits patient prognosis.

## Data Availability

The original contributions presented in the study are included in the article/Supplementary Material, further inquiries can be directed to the corresponding author.
